# Different relationship of magnocellular-dorsal function and reading-related skills between Chinese developing and skilled readers

**DOI:** 10.1371/journal.pone.0179712

**Published:** 2017-07-13

**Authors:** Jing Zhao, Hong-Yan Bi, Max Coltheart

**Affiliations:** 1 Center for Brain Science and Learning Difficulties, Key Laboratory of Behavioral Science, Institute of Psychology, Chinese Academy of Sciences, Beijing, China; 2 Department of Psychology, University of Chinese Academy of Sciences, Beijing, China; 3 Centre for Cognition and its Disorders, Macquarie University, Sydney, Australia; University of Akron, UNITED STATES

## Abstract

Previous studies have indicated that the relationship between magnocellular-dorsal (M-D) function and reading-related skills may vary with reading development in readers of alphabetic languages. Since this relationship could be affected by the orthographic depth of writing systems, the present study explored the relationship between M-D function and reading-related skills in Chinese, a writing system with a deeper orthography than alphabetic languages. Thirty-seven primary school students and fifty-one undergraduate students participated. Orthographic and phonological awareness tests were adopted as reading-related skill measurements. A steady-pedestal paradigm was used to assess the low-spatial-frequency contrast thresholds of M-D function. Results showed that M-D function was only correlated with orthographic awareness for adults, revealing an enhancement with reading development; while being related to phonological awareness only for children revealing a developmental decrement. It suggested that the mechanism responsible for the relationship between M-D activity and reading-related skills was affected by the characteristics of literacy development in Chinese.

## Introduction

Reading begins with basic visual processing. In the primate visual system, there are mainly two parallel processing streams, that is, the magnocellular-dorsal (M-D) pathway and the parvocellular-ventral (P-V) pathway. The M-D pathway passes from the retina through the M layers of the lateral geniculate nucleus to primary visual cortex, further projecting preferentially to the dorsal stream of visual cortical areas, including visual motion areas (V5/MT), posterior parietal cortex (PPC), and orbitofrontal cortex [1, 2]. The M-D pathway responds preferentially to visual stimuli with low spatial frequency or high temporal frequency, such as stimuli with blurred contours or fast movement [1]. It has been reported that visual M-D functions are closely associated with reading processes [3, 4]. Various studies have shown that individuals with developmental dyslexia (DD) exhibit M-D deficits [5–9], and M-D function training for dyslexics can improve their reading-related skills [10–13]. Recent research, including a study with reading level controls, a longitudinal study and training studies on both adults and children with dyslexia, has consistently suggested a causal link between an M-D deficit and developmental dyslexia [14].

Great efforts have been made to explore the underlying mechanism of the relationship between M-D function and reading [4, 15]. Some researchers have argued that M-D function might play a role in global orthographic recognition [15–17]. Relevant studies from different language systems [1, 16, 17] reported that the M-D pathway was responsible for quickly generating a first-pass global image of the word (character), which would facilitate the global recognition of this word (character) and further affected its visual-orthographic processing. Moreover, the M-D pathway has been suggested to be involved in the top-down manipulation to the brain areas over the P-V stream functioning in visual word form processing (e.g. inferior temporal cortex) [18–20], which may also underlie the relationship between M-D function and orthographic processing. Other researchers have pointed out that M-D function may play a role in phonological processing [8, 21]. The studies from alphabetic languages [8, 22–25] and Chinese [21] found that the M-D function engaged in letter (radical) position decoding because it controls visual guidance of attention, which would mediate the ability to rapidly identify letters (radicals) and their order in words (characters) [8]. And this would affect the phonological processing and in turn exerts an influence on the overall reading [21–25].

Since orthographic and phonological skills play important and special roles at different stages of literacy development [26, 27], it has been suggested that the relationship between M-D function and these reading-related skills may vary with stage of reading development [28]. In the context of alphabetic languages, both behavioral [29–32] and neurophysiological [5, 33, 34] studies have shown that M-D function is mainly related to orthographic skills in developing readers while being related to sublexical phonological processing in skilled readers. Orthographic processing plays a key role in the initial phase of learning to read [35]. For alphabetically-written languages, phonological coding gradually replaces orthographic processing as grapheme-to-phoneme correspondence (GPC) rules are acquired during the course of learning to read [36, 37].

A difference in orthographic depth between writing systems might also exert an influence on the relationship between M-D function and reading-related skills. It has been reported that M-D function is related to orthographic skills in English children aged from 7 to 12 years [38, 39]. In contrast, a relationship between M-D function and phonological processing was found in German developing readers aged from 8 to 11 years [40]. The main difference between English and German is orthographic depth: English is considered to have a deeper orthography than German [41]. Previous studies have suggested that beginning readers prefer to use logographic processing before they acquire GPC rules [36, 37]. If the transition point from depending on logographic processing to relying on GPC route is later for deep orthographies such as English, developing readers in English would show a relationship between M-D function and orthographic skills for a longer time. In contrast, if acquisition of GPC rules is earlier in languages with shallow orthography (e.g. German), M-D function would be related to sublexical phonological skills at an earlier stage of development.

In contrast to alphabetic languages, Chinese does not have GPC rules because it does not have graphemes; it has instead a logographic writing system (i.e. a particularly deep orthography). So it is particularly interesting to explore whether there is a developmental transition concerning the relationship between M-D function and reading-related skills in Chinese, in order to understand the role of writing systems in this relation. Relevant research has indicated that performance in a coherent motion detection task is related to Chinese orthographic ability for both children [6, 42] and adults [18]. However, there is other research in which M-D function was measured by a temporal order judgement task [21] which has shown that Chinese children’s M-D function is related to phonological awareness. The different findings might be due to the different visual tasks: it has been demonstrated that visual sensitivity in the coherent motion detection task might be important for global processing in orthographic processing [39]; while visual serial processing in the temporal order judgement task might be implicated in the mapping between graphemes and phonemes in phonological awareness [21].

Most of the relevant research has used the coherent motion detection and temporal order judgement tasks as specific measurements of M-D function. However, the validity of these visual tasks has been questioned. The coherent motion detection and temporal order judgement tasks would mainly induce cortical activity in the M-D pathway, such as activation in MT areas and PPC where the M-D input might interact with visual inputs through parvocellular-ventral pathway, so these two tasks may not be pure measurements of M-D function [38]. It has been suggested that visual processing is highly segregated throughout the subcortical portion of M-D pathway [43], so that measurement focusing on subcortical processing could better separate the M-D pathway from other visual pathways [44]. Previous studies have usually employed simple visual stimuli such as sinusoidal gratings with low spatial frequency to induce subcortical activity in the M-D pathway [2, 43]. Based on the specificity in the spatial frequency and contrast gain properties of the M-D pathway, Pokorny and Smith [45] designed the steady-pedestal paradigm to specifically assess the function of M-D pathway, and this paradigm is now regarded as the preferred method for assessing visual M-D function [46, 47]. The steady-pedestal task measures luminance contrast threshold for detecting a grating of low spatial frequency, which is presented in a brief pulse on a steady luminance pedestal [45]. Therefore, the present study explores the relationship between M-D function and reading-related skills in Chinese by using the steady-pedestal paradigm to specifically assess visual M-D function. Both primary school students and undergraduate students were recruited in order to examine any difference in this relationship between developing and skilled readers.

## Materials and methods

### Participants

Thirty-seven children from the 3^rd^ to 5^th^ grades (fifteen males) and fifty-one undergraduate students (twenty-three males) participated in the present study. All participants were right-handed Mandarin speakers with normal or corrected-to-normal vision. For the children, written consent was obtained from parents or teachers; as to the adults, written consent was obtained from each participant prior to the experiment. The study was approved by the ethics committee of the Institute of Psychology, Chinese Academy of Sciences, China.

### Psychometric tasks

#### Reading-related tests

Orthographic and phonological awareness tests were adopted as the reading-related skill assessments. The order of these tests was randomized across participants. Details of each test were as below.

**Fig 1:**
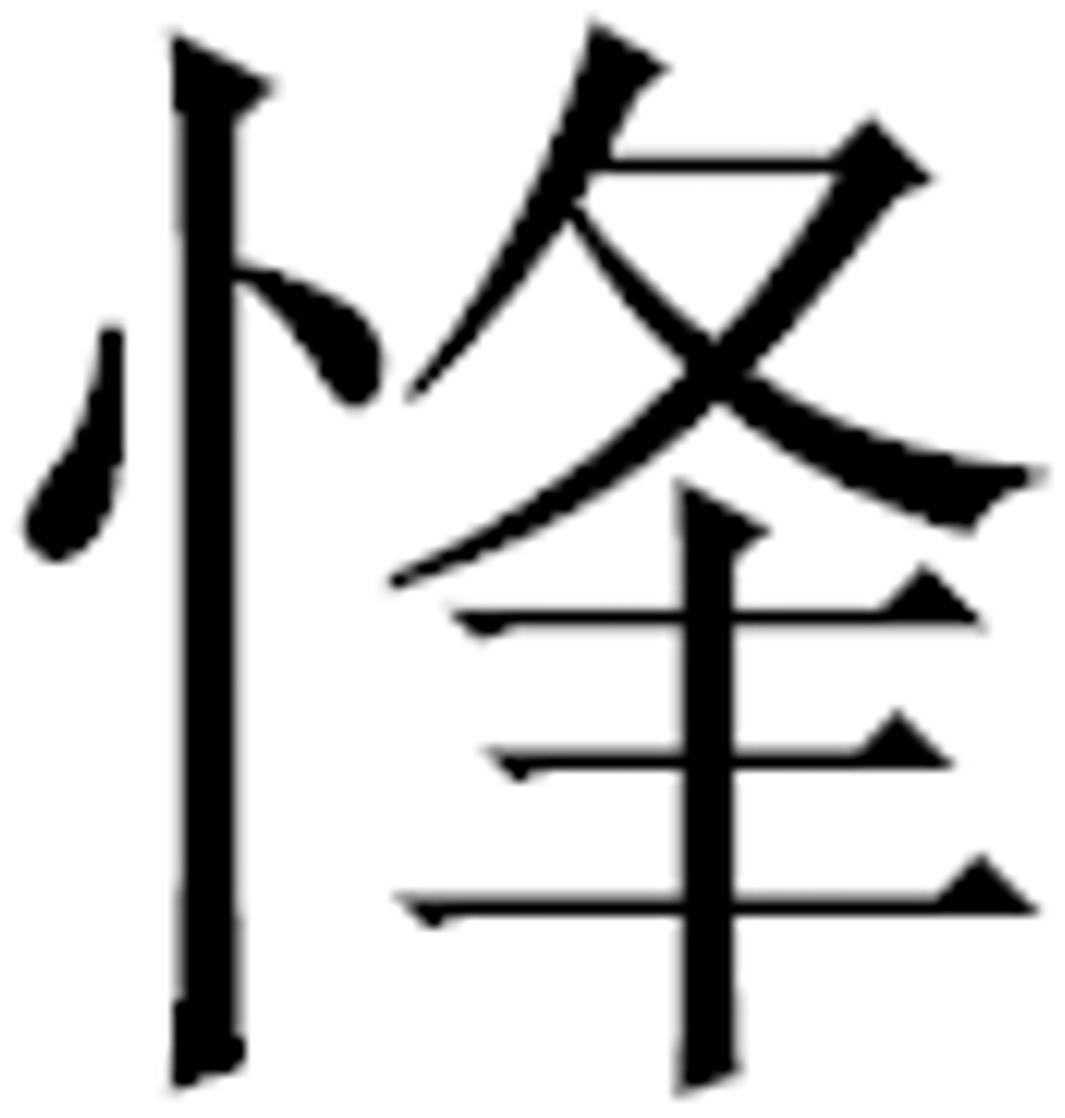
A sample of pseudocharacter.

**Fig 2:**
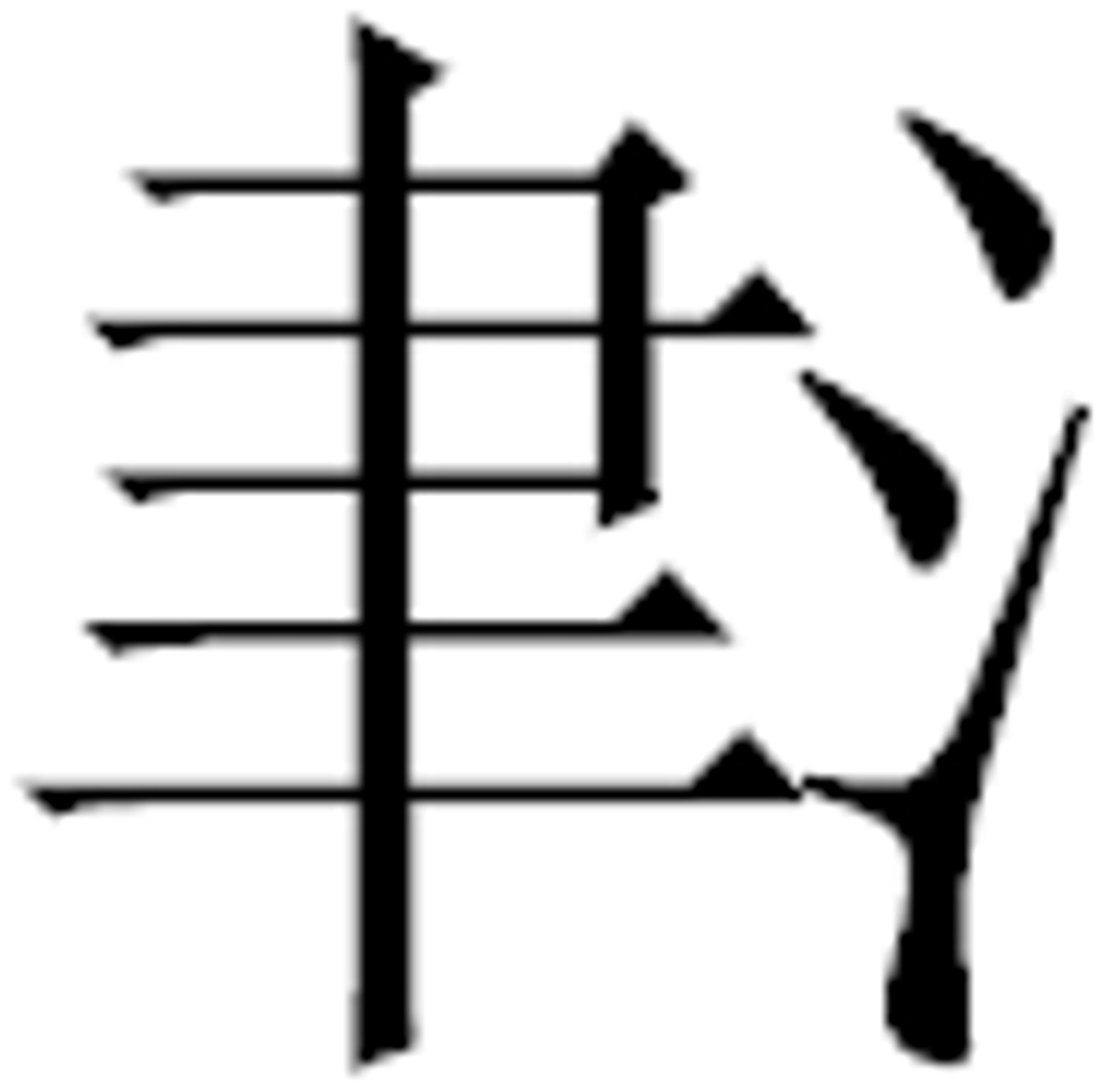
A sample of non-character.

(a) Orthographic awareness (OA) test. The OA test was the same as that used in the study of Qian et al. [18]. This test consisted of 40 real characters, 20 pseudo-characters, and 20 non-characters. The real characters in the OA test were all semantic-phonetic compounds, in which the phonetic and semantic radicals followed the orthographic rules with locating in correct positions. Some semantic radicals (e.g. “忄”, “氵”, “犭”, “扌”) are always in the left part of the character; while some other semantic radicals mostly locate in the right part of the character, such as “攵”, “刂”, “彡”. Similarly, some phonetic radicals such as “夆”, “莫”, “聿”, “青” always locate in the right, while some phonetic radicals (e.g. “害”, “乘”, “壴”) are always in the left of the character (for details of the Chinese orthography, see [6]). For example, the real character “情”(with the pronunciation /qing2/, and the number represents its tone; the character’s meaning is *feeling*) has the semantic radical “忄” in the left side and the phonetic radical “青” (/qing1/) in the right side. As to the pseudo-characters in the present test, their visual configuration followed the orthographic rules regarding radical position. For example, the pseudo-character[Fig 1], with the semantic radical “忄” in the left and the phonetic radical “夆” in the right. However, the visual configuration of the non-characters did not follow the orthographic rules of Chinese characters, such as a non-character [Fig 2] with misplacing radicals in which the semantic radical “氵” was erroneously placed in the right side and the phonetic radical “聿” was erroneously put in the left side of the item. The task was computerized, and each item was presented in isolation in the center of the computer screen. Participants were required to judge whether a presented item was a real character or not as accurately and quickly as possible, and the total accuracy was recorded which was regarded as the final score. The internal consistency reliabilities were 0.72 for child participants and 0.76 for adults, which were computed by Kuder-Richardson Formula 20.

(b) Phonological awareness (PA) test. An odd-one-out paradigm [13, 18] was adopted for this test. It measured sensitivity to the onsets, rimes and tones of spoken Chinese monosyllabic words, which belongs to the “broad” definition of phonological awareness as stated by Castles and Coltheart [48]. Similarly with English, Chinese pinyin also includes 26 letters. Some of these letters (or letter combinations) are always used as the initial consonant of a Chinese syllable (i.e. onset), such as “t”, “b”, “p”, “w”, “sh”, “ch”, and so on; while some of these letters (or letter combinations) are regarded as the final sound (i.e. rimes), such as “o”, “u”, “an”, “in”, “ian”. An onset and a rime make up of a Chinese syllable which may be pronounced in up to four different tones (the 1st tone: high level tone, the 2nd tone: rising tone, the 3rd tone: falling-rising tone, the 4th tone: falling tone). Different tones can give the same syllable different lexical status, and thus both the correct syllable and the correct tone for that syllable needs to be retrieved in language processing [49]. For example, a monosyllable “nian2”, its onset is “n” at the beginning, and its rime is “ian”, and it sounds with rising tone (i.e. the 2nd tone). There were 30 trials in the present test. Within each trial, three monosyllabic words (including consonant, rhyme, tone in each syllable) were presented auditorily for about 2500 ms, and then a fixed response window was following which lasted for 6000 ms. Participants were required to select the phonologically odd item from the syllable trio. The auditory stimulus for each monosyllable lasted for about 350–500 ms, and the interval between the successive syllables was about 500 ms. The auditory stimuli were spoken in a female voice which were recorded and played to the participants by Microsoft PowerPoint 2017. There were three types of oddity: onset, rime, and lexical tone. For example, for the three items of “tan4” (the number refers to tone; the same below), “tong3”, and “ji1”, the correct answer was “ji1”. Items of “tan4” and “tong3” had the same onset “t”, which was different from “ji1”. The above three items were completely different in rime and lexical tone in order to control possible confusion. There were ten trials for each type of oddity. The accuracy was recorded. If a participant made a correct response for one trial, then he/she was awarded 1 point. The maximum possible score was 30. By using Kuder-Richardson Formula 20, the internal consistency reliabilities were 0.77 for children and 0.79 for adults.

#### M-D function test

**Fig 3:**
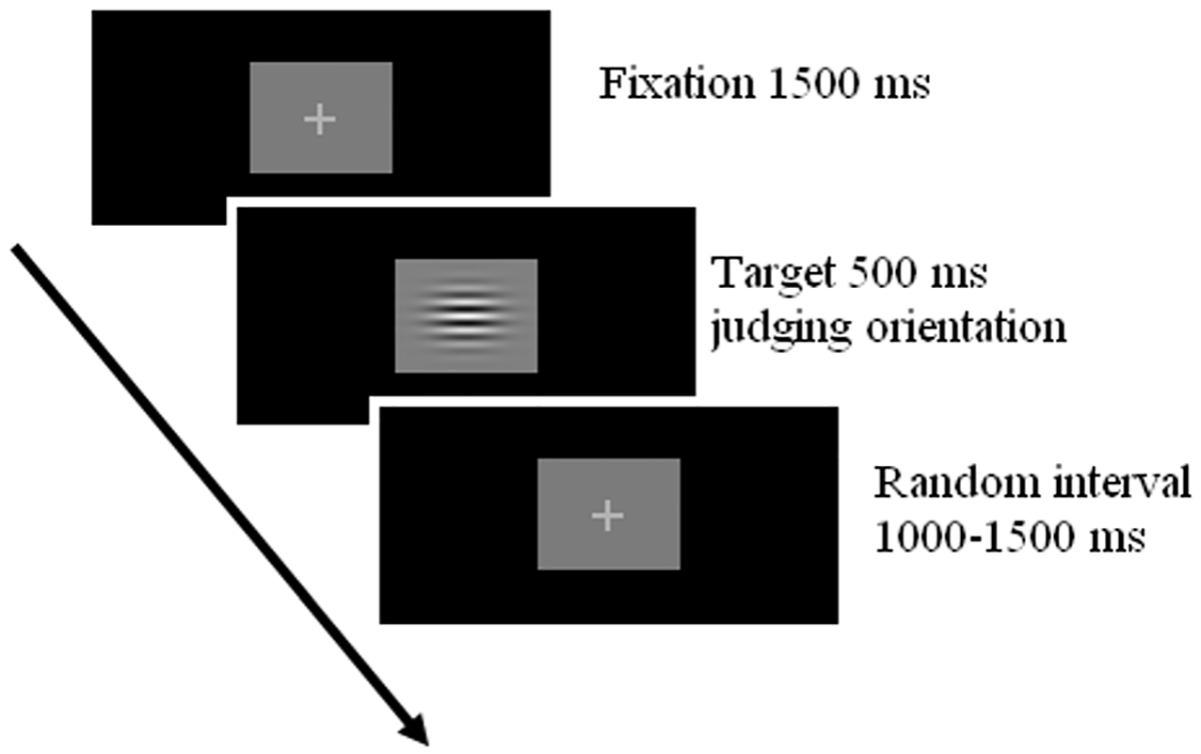
The presentation format of each trial in the test of magnocellular-dorsal function. In each trial, a fixation point was firstly displayed in the center of the screen for 1500 ms, and then a target grating appeared for 500 ms. The participants were required to press different keys to judge the orientation of the target grating, with “z” for vertical and “n” for horizontal gratings, and the response window lasted for 3000 ms. After that, a fixation point was presented in the screen center in a random interval (from 1000 ms to 1500 ms) between successive trials.

The steady-pedestal paradigm designed by Pokorny and Smith [45] was adopted to assess functioning of the M-D pathway. The experimental set-up and procedures were generally the same as those used in the study of Zhao et al. [17]. Visual stimuli were horizontal or vertical sinusoidal gratings with a visual angle of 5°×5° which were centrally displayed on a computer monitor at a distance of 50 cm. The peak spatial frequency of gratings was 0.5 cycles per degree (cpd) to activate the M-D pathway, and the luminance pedestal always stayed on the black background. Within each trial, a fixation point was presented in the center of the screen for 1500 ms, and then a target grating appeared for 500 ms. Participants were required to press different keys with two hands to judge the target orientation, with “z” for vertical and “n” for horizontal gratings, and the response window lasted for 3000 ms. A fixation point was displayed in the center of the screen in a random interval (from 1000 ms to 1500 ms) between successive trials. Fig 3 shows the presentation format of each trial.

A two-yes-one-no staircase procedure was applied to estimate the spatial-frequency contrast thresholds of vertical gratings. Details of the staircase were similar with that in the previous study of Zhao et al. [17], with a modification that the average contrast for the last six reversals was taken to estimate the contrast threshold. The contrast thresholds of the M-D function are ratios; as in previous research [17], contrast = (L_max_-L_ped_)/(L_max_+L_ped_), in which L_max_ is the maximum luminance of the grating and L_ped_ is the pedestal luminance.

There were ten practice trials before the formal experiment. The presenting procedures were programmed with Eprime 1.1. The display resolution was set at 1024×768 and the refresh rate was 62.3 Hz.

### Statistical analyses

Firstly, participant age, the scores of OA and PA tests, and low-spatial-frequency contrast thresholds of both children and adults were submitted to independent sample t tests for group comparison. And then the analysis of partial correlation was conducted within each group to measure the relationship between OA and PA, as well as to examine the relationship between M-D function and the two reading-related skills, in which the participant age was controlled. All statistics were calculated using SPSS 16.0.

## Results

The datasets of one Chinese children were discarded because her standardized score in M-D test was higher than 3 standard deviations. Data from the remaining 36 children (fifteen males) were put into further analysis.

### Descriptive statistics

Means and standard deviations of reading-related and visual M-D measures for each group are presented in Table 1. Independent sample t tests were conducted to compare the scores of reading-related and visual M-D tests between children and adults. Results showed that the performance of the adults was better than that of the children in the M-D function, orthographic awareness and phonological awareness test (Table 1).

**Table 1:** Descriptive statistics of reading-related and visual M-D measures.

Measures	Children	Adults	T values
	*M (SD)*	*M (SD)*	
**Age (years)**	9.91 (0.85)	22.71 (2.57)	33.02***
**Reading-related tests**			
OA	66.47 (4.67)	72.90 (4.19)	6.72***
PA	19.19 (3.40)	25.41 (2.98)	9.03***
**M-D function**	0.020 (0.012)	0.008 (0.003)	5.94***

Note. OA, orthographic awareness; PA, phonological awareness; M-D, magnocellular-dorsal stream.

***, *p*<0.001.

### Correlation analysis

Firstly, we conducted correlation analysis between OA and PA scores in order to ensure whether the two reading-related skills are separate from each other, meanwhile the participant age was regarded as a controlled variable. The results showed no significant correlation was found for either Chinese children (r = -0.16, p = 0.35) or adults (r = 0.16, p = 0.28).

**Fig 4:**
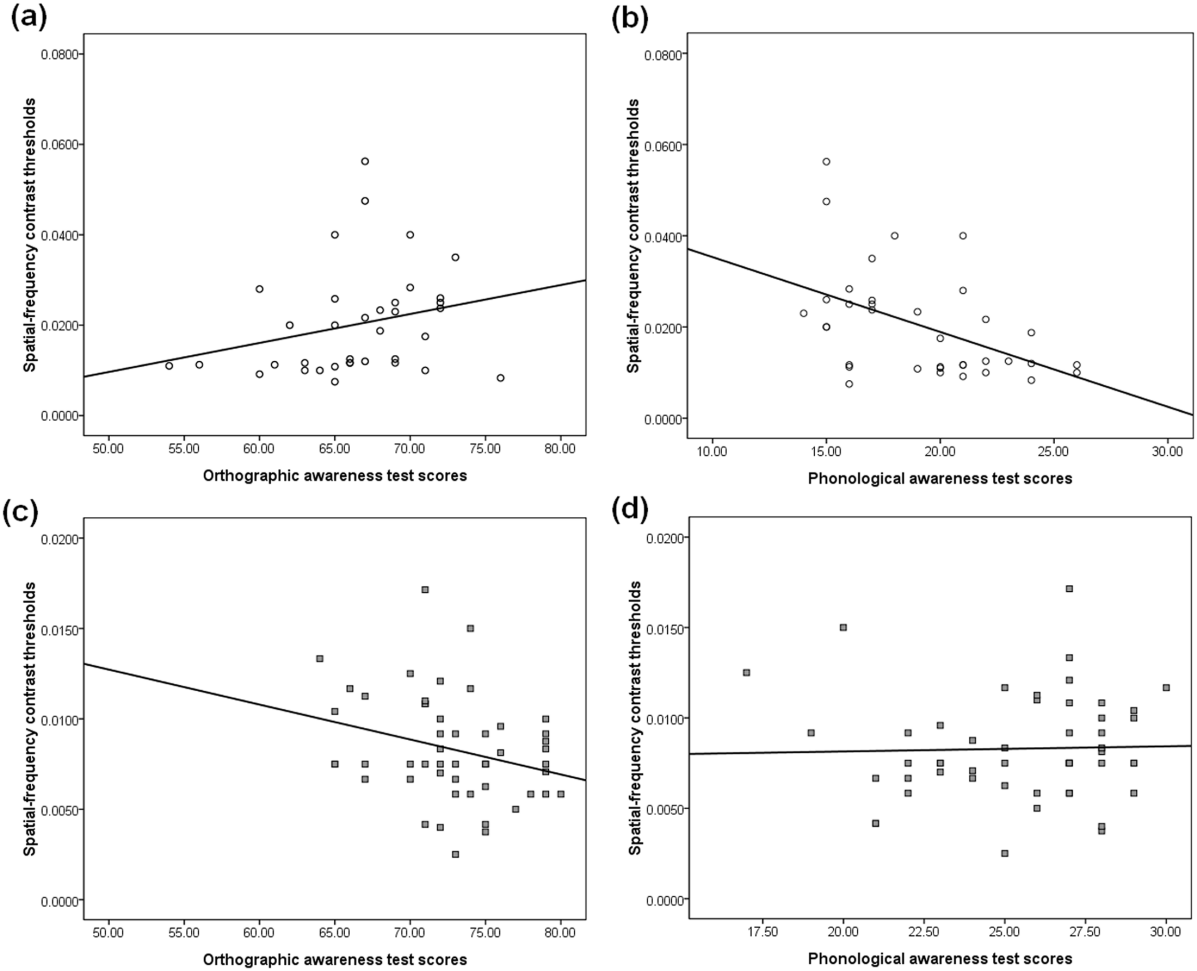
The scatter plots of the relationship between the magnocellular-dorsal function and reading-related skills. Circles correspond with the scores of children, and squares correspond with the scores of adults. (a) displays the relationship between M-D function and orthographic awareness in children, in which there was no significant correlation. (b) displays the relationship between the M-D function of children and their phonological awareness, in which the M-D function was negatively correlated with phonological awareness. (c) shows the relationship between M-D function and orthographic awareness in adults, in which a negative correlation was observed. (d) displays the relationship between M-D function of adults and their phonological awareness without significant correlation.

Secondly, analysis of partial correlation was conducted to measure the relation between M-D function and reading-related skills when the variable of participant age was controlled (Fig 4).

For the Chinese children, the spatial-frequency contrast thresholds were negatively correlated with PA scores [r = -0.47, p = 0.004], where lower contrast thresholds corresponded to higher scores in the PA tests. Whereas, the scores in M-D test was not correlated with the OA scores [r = 0.25, p = 0.14].

For the Chinese adults, the results of partial correlation analysis showed that the contrast thresholds of M-D pathway were negatively correlated with OA test scores [r = -0.28, p = 0.05], where lower contrast thresholds corresponded with the higher OA test scores. There was no significant correlation with PA scores [r = 0.04, p = 0.78].

## Discussion

### The difference in the relationship of M-D function and Chinese orthographic skills between children and adults

The present result showed an association between M-D function and orthographic skill only in Chinese skilled readers, which is consistent with previous studies in Chinese [17, 50, 51]. It has been reported that the low-spatial-frequency condition could contribute to the global processing of characters’ visual/orthographic structure in Chinese adults [50, 51], revealing the relationship between M-D function and orthographic skills in Chinese. Moreover, in the present study, the correlation was absent in children while significant in adults, seemingly revealing an enhancement of this relationship with reading development. This developmental pattern is at odds with that found in alphabetic studies [39, 52, 53]. In alphabetic languages, it has been argued that there is a developmental decrement in the relationship between M-D function and orthographic skills [39, 52, 53]. The difference in developmental patterns across writing systems might be associated with different characteristics of these writing systems. With literacy development in alphabetic languages, the acquisition of GPC rules would reduce the role of orthographic skills in reading [35]. The visual configuration of a Chinese character is more complex than that of an alphabetically-written word, and previous studies have suggested that the orthographic processing played an important and sustained role in Chinese reading acquisition [54–56].

Some neuroimaging findings provided a possible explanation for the closer relationship between M-D function and Chinese orthographic skills for adults as compared to children in our study. Some research has indicated that the M-D pathway may be involved in the top-down amplification of character detectors in brain areas over the P-V stream, such as inferior temporal cortex [43, 57], which are closely associated with orthographic processing [58]. It has been reported that the functional connectivity between brain areas over the M-D pathway and the P-V pathway might be more robust in skilled readers than developing readers in Chinese reading [59, 60], which might result in a closer association between M-D function and orthographic processing for skilled readers in Chinese compared to developing readers.

### The difference in the relationship of M-D function and Chinese phonological skills between children and adults

The present findings showed a significant correlation between M-D function and phonological awareness in Chinese children but not in adults, which is consistent with previous research in Chinese [21, 61]. These findings consistently indicate a decrement in the relationship between M-D function and phonological skills as Chinese reading development progresses. However, the developmental pattern in this relationship is different from that in alphabetic languages, in which no developmental decrease was observed [39, 61, 62]. The different patterns in the relationship seemingly depends on differences in the characteristics of reading development between writing systems. With reading development in alphabetically-written languages, the preference of readers turned from relying on logographic processing to dependence on phonological skills [36, 37]. In Chinese, primary school students are taught Chinese mainly through Pinyin (which expresses spoken Chinese using the Roman alphabet). Pinyin plays a special role in bridging the gap between speech and the written form of Chinese characters for beginning readers [26]. With the increase in reading experience, the direct connections between visual forms and corresponding meanings of Chinese characters would be well established, and the utilization of pinyin in reading procedure would be gradually diminished [63], with the decrease in the influence from phonological skills [26, 63].

The present phonological task may involve conjuring up visual representations of the auditorily-presented syllables via a sound-to-spelling conversion process, in which participants perform the PA tasks by manipulating these pinyin representations. Some researchers have pointed out that the M-D pathway is essential in creating the visual images of spoken stimuli [62]. Neuroimaging studies reported that processing linguistic information in the auditory mode would induce the occipito-temporal activity which was responsible for processing the visual form of scripts, and it has been found that the activation was stronger in children than in adults [64, 65]. Their neuroimaging finding revealed the greater activation of relevant visual/orthographic representations of spoken stimuli in children as compared to adults [64, 65]. Accordingly, it could be proposed that developing readers’ performance in the PA test (i.e. in auditory mode) might depend on the generation of pinyin representations of auditorily-presented syllables [24, 66, 67], which might result in a close relationship between their M-D function and phonological awareness due to the possible role of M-D pathway in creating the visual images of spoken stimuli [62]. While for the Chinese adults, making a decision in the PA test might rely less on the relevant pinyin representations of the syllables, and consequently their M-D function would be not significantly related with phonological awareness.

## Conclusions

The present findings of a close relationship between M-D function and Chinese reading-related skills contribute to making clear a possible role of the M-D pathway in Chinese reading, providing further evidence for the M-D deficit theory of developmental dyslexia. In detail, the present study found that the M-D function of developing readers was related to Chinese phonological skills, while the skilled readers’ M-D function was related to orthographic processing, suggesting that the relevant mechanism regarding the relationship between M-D activity and reading depends on characteristics of Chinese literacy development. However, the current findings reveal only a correlational relationship between M-D function and the reading-related skills, rather than a causal relationship. To clarify the causal role of M-D function in the reading-related skills, further studies, especially intervention and longitudinal studies, are needed.

## Supporting information

S1 File Visual and reading test scores for Chinese adults. This file includes the information of participant age, gender, as well as the data of low-spatial-frequency contrast threshold, orthographic and phonological awareness test scores.
(SAV)

S2 File Visual and reading test scores for Chinese children. This file includes the information of grade, age and gender of children. Meanwhile their scores in visual magnocellular-dorsal function test, orthographic and phonological awareness test are shown.
(SAV)
